# A simplified search strategy for identifying randomised controlled trials for systematic reviews of health care interventions: a comparison with more exhaustive strategies

**DOI:** 10.1186/1471-2288-5-23

**Published:** 2005-07-23

**Authors:** Pamela Royle, Norman Waugh

**Affiliations:** 1 Department of Public Health, University of Aberdeen, Foresterhill, Aberdeen AB25 2ZD, Scotland

## Abstract

**Background:**

It is generally believed that exhaustive searches of bibliographic databases are needed for systematic reviews of health care interventions. The CENTRAL database of controlled trials (RCTs) has been built up by exhaustive searching. The CONSORT statement aims to encourage better reporting, and hence indexing, of RCTs. Our aim was to assess whether developments in the CENTRAL database, and the CONSORT statement, mean that a simplified RCT search strategy for identifying RCTs now suffices for systematic reviews of health care interventions.

**Methods:**

RCTs used in the Cochrane reviews were identified. A brief RCT search strategy (BRSS), consisting of a search of CENTRAL, and then for variants of the word random across all fields (random$.af.) in MEDLINE and EMBASE, was devised and run. Any trials included in the meta-analyses, but missed by the BRSS, were identified. The meta-analyses were then re-run, with and without the missed RCTs, and the differences quantified. The proportion of trials with variants of the word random in the title or abstract was calculated for each year. The number of RCTs retrieved by searching with "random$.af." was compared to the highly sensitive search strategy (HSSS).

**Results:**

The BRSS had a sensitivity of 94%. It found all journal RCTs in 47 of the 57 reviews. The missing RCTs made some significant differences to a small proportion of the total outcomes in only five reviews, but no important differences in conclusions resulted. In the post-CONSORT years, 1997–2003, the percentage of RCTs with random in the title or abstract was 85%, a mean increase of 17% compared to the seven years pre-CONSORT (95% CI, 8.3% to 25.9%). The search using random$.af. reduced the MEDLINE retrieval by 84%, compared to the HSSS, thereby reducing the workload of checking retrievals.

**Conclusion:**

A brief RCT search strategy is now sufficient to locate RCTs for systematic reviews in most cases. Exhaustive searching is no longer cost-effective, because in effect it has already been done for CENTRAL.

## Background

Literature searching for systematic reviews of interventions in health care has been largely based on finding all randomized controlled trials (RCTs), as this study design is considered the gold standard. However RCTs make up only a very small proportion of all the articles included in bibliographic databases, and so the problem for systematic reviewers has been to devise a search strategy which is sensitive enough to find all the RCTs, but specific enough not to bury them in a large number of other unwanted retrievals needing to be manually excluded.

Two developments have facilitated searching for RCTs. The first is CENTRAL (the Cochrane Central Register of Controlled Trials), the world's most comprehensive database consisting exclusively of controlled clinical trials. It currently contains over 425,000 citations. The majority of the trials in CENTRAL have been identified through systematic searches of MEDLINE and EMBASE. The first two phases of a three phase highly sensitive search strategy (HSSS) have been used to search MEDLINE [1]. CENTRAL also includes citations to reports of controlled trials that are not indexed in MEDLINE or EMBASE, derived through searches of other bibliographic databases, and extensive hand searching.

Because identification has relied solely on the titles and, where available, the abstracts, some relevant trials may not have been identified. Therefore, the Cochrane Handbook says *"it may still be worthwhile for reviewers to search MEDLINE using the Cochrane highly sensitive search strategy and to obtain and check the full reports of possibly relevant citations" *[2]. However, this strategy is complicated to run, and may require time-consuming screening of abstracts, and perhaps of full articles.

The second development is the CONSORT statement, introduced in 1996 [3], and since revised [4]. CONSORT comprises a 22 item checklist and a flow diagram to help improve the quality of reports of RCTs, and has been endorsed by prominent medical journals There is evidence from a comparative before-and-after evaluation that there has been an increase over time in the number of CONSORT checklist items included in the reports of RCTs [5].

Item 1 on the CONSORT checklist recommends that the method in which participants were allocated to interventions (e.g., "random allocation", "randomized" or "randomly assigned") is described in the title and abstract. This allows instant identification of RCTs, and should help ensure that a study is appropriately indexed as an RCT in bibliographic databases.

As the Cochrane Collaboration has already done exhaustive work to ensure that CENTRAL is as complete as possible, we wanted to examine whether this eliminates the need for individual reviewers to run the HSSS, and the effectiveness of replacing this with a simplified search strategy.

### The aims were

1. To determine the effect on the results of Cochrane reviews of using a brief RCT search strategy (BRSS).

2. To examine the change in use of variants of the word random in the title or abstract of journal articles, pre- and post-CONSORT.

## Methods

All reviews new to the Cochrane Database of Systematic Reviews in the Cochrane Library 2004 issue 2 were selected. Those that stated that they were considering only RCTs, and which found at least one RCT, were selected.

All trials included in each review were identified from the section 'References to studies included in this review'. Each trial was checked to determine whether it was indexed in CENTRAL (on Cochrane Library 2004, issue 2), and then MEDLINE. If not in either of these databases, EMBASE was checked.

The full bibliographic records of all trials that were in either MEDLINE or EMBASE (using the OVID interface) were examined to determine whether random$ was in any field. (Random$.af. means a search of variants of random in all fields, where $ is truncation symbol).

The full papers of any trials that were: 1) not in CENTRAL, or 2) did not have random$ in any field in the bibliographic record, were obtained and checked to see whether they were actually RCTs. All non-English articles were translated, apart from those in Japanese and Chinese, as resources were not available.

The impact of omitting the RCTs not found with the BRSS was quantified, using Review Manager 4.2.7. The forest plots for the meta-analyses were reproduced, both with and without data from the missing trials, and the results compared to see if omission would make any important difference. The differences could theoretically include;

a) There might be less, or no data left, for some outcomes.

b) There could be a different result; no benefit over comparator, or vice versa.

c) There could be the same result, but with a different effect size.

d) There could be the same result and effect size, but with a wider confidence interval, and possibly loss of statistical significance.

In summary, the BRSS would involve: 1) searching CENTRAL, and 2) supplementing that with a search of MEDLINE and EMBASE, using a search of 'random$.af.' to pick up trials not in CENTRAL.

## Results

There were 78 new reviews, of which 57 met the inclusion criteria. They cited a total of 920 trials; 79% (725) were journal articles. The remaining 21% were from the grey literature, 80% of these being conference abstracts).

Twenty one reviews were excluded from our study; 14 because they did not find any RCTs that met their inclusion criteria, and seven because they included other study designs in addition to RCTs.

### Determination of the proportion of journal articles found using the BRSS

It was found that 93.3% (677) of the 725 journal articles included in the systematic reviews were in CENTRAL. It was assumed that these were all RCTs, but this was not checked due to the large numbers involved (and it was not relevant to the aims of the study). This left 48 journal articles not indexed in CENTRAL.

The full texts of all 48 of these articles (apart from the four in Japanese and five in Chinese) were translated and checked. It was found that 40 were RCTs, and the remaining eight used non-RCT study designs. (It was assumed that the untranslated articles were RCTs). Eleven of the 40 RCTs were found by searching MEDLINE or EMBASE with 'random$.af'. Therefore, 29 (4%) of RCTs would not have been found with the BRSS.

### Details of the 29 RCTs not found by the BRSS

The 29 trials were distributed over 12 reviews. Ten were in MEDLINE, 11 in EMBASE only. Twelve were non-English language.

In two reviews [6,7], each with one missing trial, the data used in the meta-analyses were available in two other papers; both were in CENTRAL, and included in the reviews. Zhang 1990 [8] was confirmed (by authors of the review) to be the same trial as Chang 1996 [9]. The data in the Stensrud trial [10] was also reported in another paper by Stensrud [11].

Therefore, this leaves 27 missing journal articles, in 10 separate reviews that contain at least one trial with data not found by the BRSS. Table 1 shows the detail of these trials and the impact of excluding them from the reviews.

**Table 1:** Summary of impact of excluding trials not found with brief RCT search.

**Topic**	**No RCTs included**	**Details of RCTs not found with BRSS**	**Effect on review of excluding the RCTs not found**
Lymphoedema of the limbs [20]	4	1. Kasseroller (1996) [21]	Kasseroller study involved in 2 of 17 meta-analyses; exclusion makes no significant difference to outcome of either meta-analysis.
Ventricular pacemakers [22]	34	1. Davis (1985) [23]	Davis study included in 1 of 12 meta-analyses; exclusion makes no significant difference.
Early intervention for psychosis [13]	7	1. Linszen (1998) trial comprised 5 papers; 2 not found with brief search. a) Linszen (1998a) [24] b) Linszen (1998b) [25]	Linszen study included in 1 of 12 meta-analyses. Excluding Linszen would mean that there would be no data for this outcome.
Interventions for impetigo [15]	60	1. Arata (1989a) [26] 2. Arata (1989b) [27] 3. Bass (1997) [28] 4. Koranyi (1976) [29] 5. Moraes Barbosa (1986) [30] 6. Park (1993) [31] 7. Pruksachat. (1993) [32] 8. Sutton (1992) [33] 9. Tamayo (1991) [34]	The 9 trials were included in 8 of the 19 meta-analyses in this review. Outcome 1: excluding Sutton, Bass and Tamayo studies individually made no significant difference. The removal of all 3 together causes meta-analysis to lose statistical significance. Outcome 2: excluding Bass, Park, Koranyi; no significant change Outcome 3: excluding Park; no significant change Outcome 4: excluding Arata 1989a; no significant change Outcome 5: excluding Pruksachat.; no significant change Outcome 6: excluding Arata 1989b; only trial providing data to this outcome, so no data now available. Outcomes 7 & 8: Moraes-Barbosa 1986 is the only trial providing data for both outcomes, so no data now available.
Adherence to treatment in patients with high blood pressure [12]	38	1. Gabriel (1977) [35] 2. Hamilton (1993) [36] 3. Kerr (1985) [37] 4. Morisky (1985) [38] 5. Rehder (1980) [39]	58 different interventions were tested on 15519 patients. Review did not do meta-analysis due to heterogeneity between studies in terms of interventions and the methods used to measure adherence. Missing trials were all small studies of poor methodological quality. Their exclusion would have little impact on the final conclusions.
Preventing infection in nephrotic syndrome [14]	5	1. Dou (2000) [40] 2. Li (2000) [41] 3. Zhang (2000) [42] 4. Tong (1998) [43]	The 4 missing trials were included in 3 of 4 of the meta-analyses. Outcome 1: Excluding Dou and Tong; no significant change. Outcome 2: Tong was the only trial providing data, so no data now available. Outcome 3: Zhang was the only trial providing data, so no data now available. Outcome 4: Li was the only trial providing data, so no data now available.
Probiotics for treating infectious diarrhoea [17]	25	Sugita (1994) [44]	Sugita included in 7 of 27 meta-analyses in review. Exclusion of Sugita makes no significant difference to any of the clinical outcomes. The only outcome to lose statistical significance is not a clinical outcome, but a sensitivity analysis based on a methodological characteristic (blinding).
Psychological interventions for coronary heart disease [16]	56	1.Gallacher (1997) [45] 2. Mitsibounas (1992) [46]	1. Gallacher was included in 8 of 28 meta-analyses. In six, omission makes no difference. In 2, confidence intervals are much wider; in the one of these statistical significance is lost. 2. Mitsibounas was included in 5 of 28 meta-analyses. Excluding Mitsibounas makes no significant difference in four outcomes. In the other, it cause causes the meta-analysis to lose statistical significance
Insulin analogues versus human insulin [47]	43	Iwamoto (2001) [48]	Iwamoto in 4 of the 8 meta-analyses. Exclusion of Iwamoto makes no significant difference to any of the outcomes.
Tramadol for neuropathic pain [49]	5	Leppert (2001) [50]	Leppert not used in either of 2 meta-analyses in the review. The review says: 'Given the small (even if unknown) number of subjects and the non-blinded nature of the trial it is probably not possible to draw and conclusions about their relative efficacy from this study.

Only one review did not do a meta-analysis[12] The nine remaining reviews did a total of 129 meta-analyses of various outcomes. In five out of the 10 reviews the missing trials made no significant difference, in that there were no clinically significant changes in effect size, nor any change in whether results were statistically significant or not. Hence, there was some difference in only five reviews.

### The impact of the 'missing trials' being excluded from the reviews

Two consequences of missing data were found:

1) In three reviews [13-15], the missing trials were the only ones providing data for seven (of a total 35) outcomes, so no data were available for these seven outcomes. In a fourth review [16], omission of a trial would lose 92% of patients for two outcomes out of 28, but this did not change the significance or direction of the result.

2) In three reviews [15-17], the meta-analyses lost statistical significance due to the wider confidence intervals, for three of 74 outcomes, but one of these was not a clinical outcome.

### Comparison of retrieval of HSSS and random$.af. in MEDLINE

For the period 1966 to October, 2004, the HSSS search strategy retrieved 2,505,742 records in MEDLINE, compared to 'random$.af.', which retrieved 399,208 records. Therefore, only 16% of the number of records were found using 'random$.af.', compared to HSSS.

### CONSORT and the change over time in the proportion of RCTs with random$ in title or abstract

**Figure 1:**
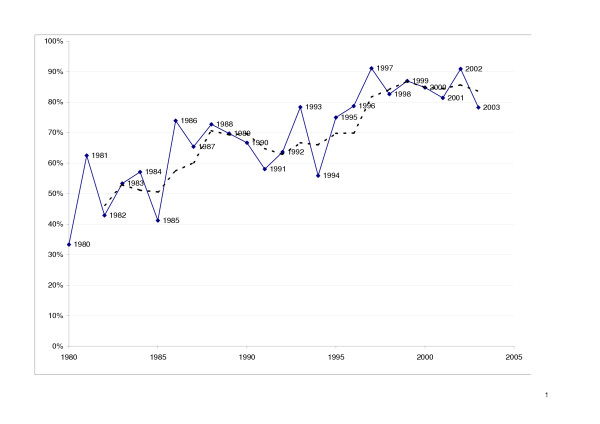
Change in the proportion of RCTs with 'random$' in the title or abstract (with three year moving average trendline added).

Using the 725 journal articles in our sample, we compared the frequency of random$ in the title or abstract between pre-CONSORT (published up to 1996) and post-CONSORT trials (published from 1997 onwards). Figure 1 shows the change in the proportion of RCTs with 'random$' in the title or abstract (with three year moving average trendline added).

In the post-CONSORT years, 1997–2003, the term appeared in 85% of the titles or abstracts, compared to 68% in the seven years before CONSORT (1990–96); mean difference was 17% (95% CI 8.3 to 25.9%; p = 0.001). If a longer pre-CONSORT period is used, 1980 to 1996, the proportion is similar at 62%.

## Discussion

Using the BRSS, rather than the much more exhaustive HSSS, to retrieve RCTs in journal articles for Cochrane reviews, affected only a very small percentage of total outcomes of a few reviews, and made no important difference to the conclusions of these reviews. The most affected review had four of the five included trials published in Chinese [14].

The CONSORT statement appears to be associated with a significant increase in the frequency of random in the titles and abstracts of journal articles. Whether this is directly due to CONSORT, or whether CONSORT simply accelerated a pre-existing trend, and raised the general awareness amongst authors and editors for better reporting of RCTs, is uncertain. This improved description of RCTs by authors should mean that all RCTs are indexed with the appropriate publication type in MEDLINE, and also result in more sensitive retrieval of RCTs using the BRSS.

The BRSS had a sensitivity of 96% for RCTs in journal articles. Compared to the HSSS, the BRSS reduced the MEDLINE retrieval by 84%. This would represent a major time and cost saving in manual screening.

The strengths of this study include firstly that we used Cochrane reviews, as they approximate the 'gold standard' in searching, due to the requirement for exhaustive searches. Hence we can be fairly certain that we were starting with as comprehensive set of included trials as possible. Secondly, we quantified the impact of omitting trials not found with the simplified search.

A weakness of this study was that we could not check whether the RCTs would have been in CENTRAL at the time the searchers did their searching, and hence eliminate the possibility that some RCTs were first identified by the reviewers, and then passed to CENTRAL. However, given the extensive searching routinely done for CENTRAL, it is highly likely most RCTs would be identified sooner or later, and therefore be included in a subsequent update of the review.

A range of subject areas was included, which helps with generalisability, though it may decrease power in any one subject area. However, a recent study on identifying quality RCTs in pain relief gives general support to our findings [18]. It investigated the efficiency of the search strategy DBRCT.af., ("double-blind," "random," or variations of these terms) in MEDLINE and EMBASE, and was found have a sensitivity of 97%.

This study focused only on a simplified search strategy for retrieving RCTs in journal articles, since these made up the vast majority of the references used in Cochrane reviews, and are most important in terms of the quality and quantity of assessable data available. By contrast, most grey literature (the vast majority of which is meeting abstracts) gives very limited data. However, CENTRAL includes many grey literature trials, so these will be identified with the BRSS.

There are currently over 2200 Cochrane reviews, and these will need maintaining in the future. Authors may be encouraged to update their reviews, if they can be confident they can identify RCTs comprehensively with a simple search. The simplified search may also be useful for those doing reviews in a tight timescale, or by clinicians who just want a rapid but reliable answer to a question. There is a case for the 'not quite perfect but rapid, easy and almost complete' search'.

In practice, some of the few trials missed by the BRSS were small or of poor quality, and as Egger and colleagues have reported, the last few studies found by exhaustive searching could introduce bias, by being of poor quality [19].

The issue is whether the marginal benefits of exhaustive searching justify the extra costs. When the inclusion criteria demand only RCTS, this study suggests that exhaustive searching is now, in the era of CENTRAL and CONSORT, no longer cost-effective.

## Competing interests

The author(s) declare that they have no competing interests.

## Authors' contributions

PR conceived and designed the study, and analysed the data. NW helped with interpretation and with drafting the paper. Both authors approved the final version

## Pre-publication history

The pre-publication history for this paper can be accessed here:


